# Clock genes and their genomic distributions in three species of salmonid fishes: Associations with genes regulating sexual maturation and cell cycling

**DOI:** 10.1186/1756-0500-3-215

**Published:** 2010-07-29

**Authors:** Marion I Paibomesai, Hooman K Moghadam, Moira M Ferguson, Roy G Danzmann

**Affiliations:** 1 Department of Integrative Biology, University of Guelph, Guelph, Ontario, N1G 2W1, Canada; 2 College of Veterinary Medicine, Cornell University, Ithaca, New York, USA

## Abstract

**Background:**

Clock family genes encode transcription factors that regulate clock-controlled genes and thus regulate many physiological mechanisms/processes in a circadian fashion. Clock1 duplicates and copies of Clock3 and NPAS2-like genes were partially characterized (genomic sequencing) and mapped using family-based indels/SNPs in rainbow trout (RT)(*Oncorhynchus mykiss*), Arctic charr (AC)(*Salvelinus alpinus*), and Atlantic salmon (AS)(*Salmo salar*) mapping panels.

**Results:**

Clock1 duplicates mapped to linkage groups RT-8/-24, AC-16/-13 and AS-2/-18. Clock3/NPAS2-like genes mapped to RT-9/-20, AC-20/-43, and AS-5. Most of these linkage group regions containing the Clock gene duplicates were derived from the most recent 4R whole genome duplication event specific to the salmonids. These linkage groups contain quantitative trait loci (QTL) for life history and growth traits (i.e., reproduction and cell cycling). Comparative synteny analyses with other model teleost species reveal a high degree of conservation for genes in these chromosomal regions suggesting that functionally related or co-regulated genes are clustered in syntenic blocks. For example, anti-müllerian hormone (amh), regulating sexual maturation, and ornithine decarboxylase antizymes (oaz1 and oaz2), regulating cell cycling, are contained within these syntenic blocks.

**Conclusions:**

Synteny analyses indicate that regions homologous to major life-history QTL regions in salmonids contain many candidate genes that are likely to influence reproduction and cell cycling. The order of these genes is highly conserved across the vertebrate species examined, and as such, these genes may make up a functional cluster of genes that are likely co-regulated. CLOCK, as a transcription factor, is found within this block and therefore has the potential to cis-regulate the processes influenced by these genes. Additionally, clock-controlled genes (CCGs) are located in other life-history QTL regions within salmonids suggesting that at least in part, trans-regulation of these QTL regions may also occur via Clock expression.

## Background

Rhythms in reproductive development, physiology, and behavior are found ubiquitously in organisms including prokaryotes, plants, fungi, and animals. Life-history events are temporally organized throughout the lifetime of an organism, which suggests that there is a time-keeping mechanism that is regulating these events. Daily biological rhythms (~24 hour cycle) are generated at the molecular level through autoregulatory positive and negative feedback loops of the core clock genes (e.g. *Clock*, *bmal1*, *Period*, *Rev-Erbα*, *Cryptochrome, Rora*). CLOCK or NPAS2 (Neuronal PAS-domain) proteins form heterodimers with BMAL1 (Brain and Muscle ARNT-like) proteins (CLOCK:BMAL1 and NPAS2:BMAL1) which activate transcription of *Period*, *Cryptochrome*, *Rev-erbα *and *Rora *genes by binding to E-box motifs (CACGTG) in their promoter regions. PERIOD and CRYPTOCHROME proteins form complexes that act as feedback inhibitors to repress the transcriptional action of CLOCK:BMAL1 heterodimers. Two E-box activated orphan nuclear receptor proteins REV-ERBα and RORA, inhibit and activate transcription of *bmal1*, respectively, by binding to the retinoid orphan receptor elements (RORE) in the promoter region of *bmal1 *(see [1] for a review). Because so many processes display circadian rhythms, the clock genes have been targeted as candidate genes with pleiotropic effects.

Disruption of circadian rhythm may have broad consequences at the molecular level, as previous studies indicate that 2% to 10% of all mammalian genes display circadian oscillation [2,3]. The expression of some of these genes is regulated by *Clock *exclusively, and these genes are called clock-controlled genes (CCGs). *Clock *encodes for a bHLH (basic-helix-loop-helix)-PAS (Period-Arylhydrocarbon receptor nuclear translocator-single minded) domain containing transcription factor which activates the transcription of CCGs. CLOCK acts as a histone acetyltransferase (HAT) to promote chromatin modifications in the promoter regions of these genes [4], and these genes in turn influence many physiological mechanisms downstream.

Clock genes are of interest as candidate genes that control life-history traits across different organisms because their allelic variation has been associated with reproductive traits in many organisms. For instance, *Drosophila *males with loss-of function for *Clk *(*Clock*) released less sperm than wild-types, thereby reducing reproductive fitness [5]. In female mice (*Mus musculus*), *Clock *mutations resulted in aberrant estrous cycles, subfertility [6], and higher pregnancy failure rates when compared to wild-type mice [7]. In salmonids, the *Clock *gene has been localized to genomic regions that influence reproductive events. Leder et al. [8] localized a copy of the *Clock *gene to a quantitative trait locus (QTL) region that explained 20% - 50% (dependent upon parental contribution) of the variation in spawning dates of female rainbow trout (*Oncorhynchus mykiss*). Also, O'Malley et al. [9] identified variant copies (alleles) of the *Clock *gene that differed significantly in frequency between fall vs. spring spawning Chinook salmon (*Oncorhynchus tshawytscha*). Because of the temporal nature of processes such as folliculogenesis, estrous cycles, and spermatogenesis, it is suggested that these processes are regulated by the *Clock *genes.

Similar to *Clock*, anti-müllerian hormone (*amh*) (a member of the transforming growth factor beta protein super family) has purported effects on reproductive events in mammals and various fish species. In rats, AMH has been shown to inhibit cAMP activity and prevent or severely reduce follicle stimulating hormone (FSH) activation of Cyp19a1, an aromatase responsible for the conversion of androgens to estrogens [10]. In mammals, *amh *expression in the testes causes the regression of the Müllerian ducts, which are precursors of fallopian tubes and the uterus in the female reproductive system. Besides its role in sex determination early in life, *amh *plays a role in both female and male gonad development and maintenance [11,12]. The Müllerian ducts in females eventually lose sensitivity to AMH and the ovarian granulosa cells express AMH which inhibits the expression of Cyp19a1 and luteinizing hormone (LH) receptors which in turn, negatively regulates folliculogenesis [13,14]. In males, *amh *activity prevents Leydig cell formation and steroidogenic enzyme production therefore inhibiting testes development [14]. *amh *expression in Sertoli cells in immature testes was also found to prevent spermatogenesis in mammals [15].

Sexual determination across the fishes is not solely dependent on genetic factors and the role of genetics in this process is not as clear as seen in mammals. *amh*-like genes have been described in some teleost species including, Japanese eel (*Anguilla japonica*) (BAB93107), Japanese flounder (*Paralichthys olivaceus*) (BAD37138), zebrafish (*Danio rerio*) and Atlantic salmon (*Salmo salar*) (AAU85130; AY722411; cluster TC149876 [16]). Examination of the expression patterns of *amh *in zebrafish [17], Japanese flounder [18], and Japanese eel [19], revealed sexually dimorphic expression patterns in juveniles as seen in mammals. In zebrafish, it was also shown that *amh *expression was present in both undifferentiated testes and ovaries later in development similar to what is seen in mammals [17]. As in mammals, Miura et al. [19] demonstrated that *amh *expression inhibited spermatogenesis. Similarly, in Atlantic salmon, *amh *levels appear to decrease rapidly at the onset of sexual maturation [20], and gene expression levels have also been reported to be several-fold higher in immature Atlantic salmon with a dramatic decrease seen in fish undergoing sexual maturation [21]. This evidence suggests that *amh *orthologs have similar roles across species. However, in contrast to this, *amh *expression was not detected in adult Japanese flounder ovaries [18]. Also, no sexually dimorphic expression of *amh *was observed in juvenile medaka (*Oryzias latipes*) [22]. In fact, within the Sertoli cells of adult medaka testes, high levels of *amh *expression were observed during spermatogenesis [22]. Although the function of AMH is less clearly understood in fishes, it does appear that differential expression of this gene is associated with differentiated sexual states in fishes.

Any research that investigates the molecular architecture of traits in vertebrates should take into consideration the past duplication events in this lineage. In humans (*Homo sapiens*) and mice, *Clock *and *NPAS2 *are two known paralogs which are related by the 2R genome duplication event [23]. Zebrafish and fugu (*Takifugu rubripes*) are known to have three copies of the Clock-family genes (*Clock1*, *Clock3*, *NPAS2*). Tetraodon (*Tetraodon nigroviridis*) has *Clock1 *and *Clock3 *genes, while medaka and stickleback (*Gasterosteus aculeatus*) have *Clock1 *and *NPAS2 *genes [23]. *Clock1 *and *Clock3 *are duplicates that resulted from the third round of whole genome duplication that occurred within the ray-finned fish lineage prior to the radiation of the teleost fishes (approximately 300 Mya = 3R hypothesis) [23]. An additional round of whole genome duplication (4R hypothesis) occurred in the salmonid lineage approximately 25 to 100 Mya [24], therefore we expect that for a single gene in most teleosts, gene duplicates may be found in the salmonids. Homeologs, which are chromosomal segments that arose from the most recent whole genome duplication events, have been previously identified in rainbow trout, Atlantic salmon, and Arctic charr (*Salvelinus alpinus*) [25,26]. *Clock1 *duplicates (homeologs) have also been identified in the Chinook salmon genome [27], supporting the 4R hypothesis. However, only singleton copies of *amh *have been identified in human, mouse, zebrafish, medaka, stickleback and the pufferfishes genomes, suggesting that there may be no functional duplicates in tetrapods, or even the ray-finned fishes. Currently, only a single *amh *copy has been identified in rainbow trout [28], however one may expect there to be a second copy due to the 4R duplication event.

This study details the findings on the genetic mapping of *Clock *and *amh *gene copies in three species of salmonid fishes. It was predicted that if duplicates of the Clock-family and *amh *genes occurred within these salmonid genomes then they should map to previously identified homeologous regions. Also considering the putative role of these genes in influencing life-history traits it was predicted that they should be located in the genomic regions where life-history quantitative trait loci (QTL) have been found in salmonids. Therefore linkage group locations were compared to previously identified QTL for life-history traits, such as female spawning date, maturation time, and developmental rate in rainbow trout, Atlantic salmon, and Arctic charr. As anticipated, Clock-family and *amh *gene copies were co-localized to major life-history QTL regions in these salmonid fishes. Duplicated *Clock *copies occurred within the same syntenic cluster as *amh *and comparative synteny analyses of other genes upstream and downstream to this region identified several other major genes that may be involved in sexual reproduction and cell cycling. This suggests that *Clock *genes may localize within a 'reproductive controlling block' in vertebrate genomes.

## Results and Discussion

### Characterization of Clock genes

**Figure 1:**
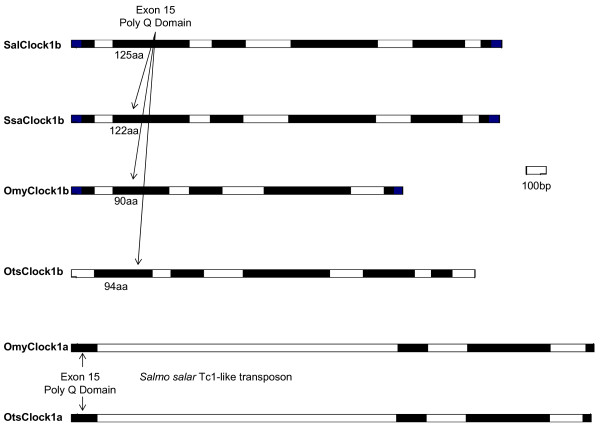
**Schematic diagram of the exons and introns of *Clock1a *and *Clock1b *genes in 4 species of salmonid fishes: Arctic charr (*Sal*), Atlantic salmon (*Ssa*), rainbow trout (*Omy*) and chinook salmon (*Ots*)**. Exons and introns are indicated by black and white boxes, respectively. The length of the protein product in number of amino acids is indicated under exon 15 for CLOCK1B proteins, which is synonymous with the PolyQ domain. The *Salmo salar *Tc1-like transposon insert located in intron 15/16 of *OtsClock1a *and *OmyClock1a *genes is indicated.

Analyses of partial *Clock1 *sequence data suggests that duplicates exist in the rainbow trout (*OmyClock1a *and *OmyClock1b*) genome while only single copies were identified in Arctic charr (*SalClock1b*) and Atlantic salmon (*SsaClock1b*) genomes. For *OmyClock1a*, 4 exons were identified that were orthologous to *Otsclock1a *exons 15 to 18 from the 3' end. Sequencing data for five exons similar to the 3' end of *DreClock1 *was obtained for *SsaClock1b *ranging from exons 14 to 18, and 6 exons for *OmyClock1b *and *SalClock1b *ranging from exons 14 to 19 (Figure 1.). As seen in Chinook salmon [27], distinct *Clock1a *and *Clock1b *sequence duplicates are evident in the rainbow trout genome, however, sequence data confirming independent *Clock1a *orthologs were not obtained from the Atlantic salmon and Arctic charr genomes. Mapping data, however, indicates that *Clock1 *duplicates do exist in Atlantic salmon and Arctic charr (discussed later), suggesting that sequence divergence between these two isoforms may be minimal in these two species.

The nucleotide sequence of the *Clock1 *duplicates, *OmyClock1a *and *OmyClock1b*, in rainbow trout were 64% identical. Coding sequences of *OmyClock1a *and *OmyClock1b *shared 90% identity which was analogous to what was seen for *OtsClock1a *and *OtsClock1b *comparisons (90%) [27]. The genomic *Clock1b *sequences from all three salmonid species were compared, and *OmyClock1b*, *SalClock1b *and *SsaClock1b *were approximately 97% similar to each other. The coding sequences of *OmyClock1b *compared to ¬*SsaClock1b *and *SalClock1b *were greater than 98% identical. The pairwise comparisons suggest that orthologous copies among species are more similar to one another than the paralogous duplicates within species, suggesting a divergence predating the speciation events within the lineage. Similar observations have been made for other gene duplicates in the salmonids [29,30].

The 64% identity between *OmyClock1a *and *OmyClock1b *was, however, unexpectedly low compared to other paralogous genes examined in salmonids. For example, duplicate genes coding for IGF and MYF share 96-97% identity [31]. The low similarity between the rainbow trout *Clock1 *duplicates was due to a large insert located in intron 15/16 which had the highest BLASTN hit to intronic regions of *Salmo salar *HoxC cluster (EU025714) with 91% identity (Figure 1). The region of similarity within the *Salmo salar *HoxC cluster was between *Hox6Caa *and *Hox8Caa *genes, which is where *Hox7Caa *was lost and replaced with a transposon [32]. The next highest hit to intron 15/16 was to the 3' end of *Salmo salar *Ras-related putative protein rab-31 (BT058970) with 89% identity. Coincidentally, these regions of rab-31 and HoxC share similarity to a Tc1-like DNA transposon DTSsa5 (EF685958) (89%) (Additional File 1). The transposon-like sequence found in *OmyClock1a *does not contain the inverted tandem repeats (ITRs) which is essential for recognition by the transposase protein for excision to occur. If excision does not occur, the transposable element may become a permanent element that is inactive. When O'Malley and Banks [27] compared the Chinook salmon *Clock1 *duplicates, *Otsclock1a *and *Otsclock1b*; they reported that intron 15/16 contained a *transferrin *insert; however this study was published prior to the annotation of the Tc1 transposons in *Salmo salar *by de Boer et al. [33].

**Figure 2:**
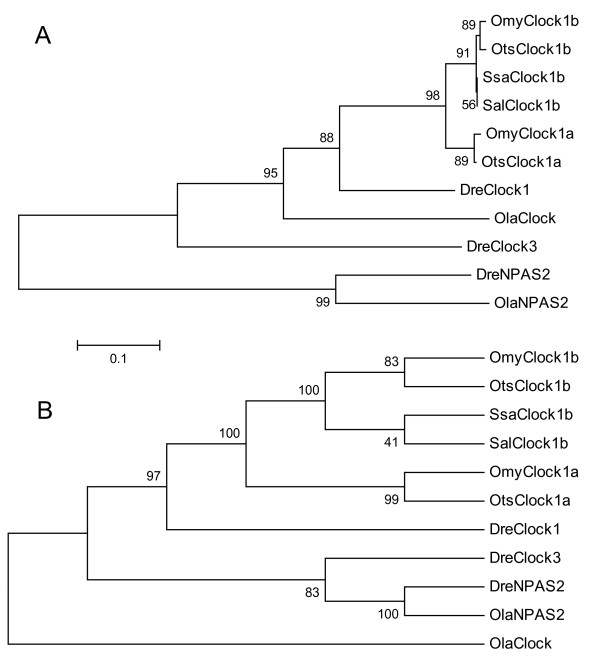
**Phylogenetic relationship of Clock nucleotide sequences obtained from Arctic charr (Sal), Atlantic salmon (Ssa), medaka (Ola), rainbow trout (Omy), and zebrafish (Dre) coding regions**. A) Neighbour-Joining. B) Maximum Parsimony. The tree was rooted to the *OlaClock *sequence. Values indicated at the nodes are the percent bootstrap values of 1000 replicates.

A single NPAS2-like sequence from rainbow trout (*OmyNPAS2-like*) showed similarity to the *NPAS2 *genes in stickleback and zebrafish. *OmyNPAS2-like *spanned exons 10 (76% identity) and 11 (84% identity) of the stickleback *NPAS2 *gene. In zebrafish, the *OmyNPAS2-like *sequence was similar to exons 11 (81% identity) and 12 (86% identity). Unfortunately, this sequence was not included in the phylogenetic analysis (Figure 2) because it covered a portion of *NPAS2 *that was not homologous to the partial salmonid *Clock1 *genes. Mapping results, however, confirm that these sequences are not *Clock1 *orthologs (discussed later).

Phylogenetic analysis revealed that the salmonid *Clock1a *and *Clock1b *duplicates were most similar to *DreClock1 *with 88% (Neighbour Joining: NJ) and 97% (Maximum Parsimony: MP) bootstrap confidence. The orthologs *OmyClock1b*, *SsaClock1b*, *SalClock1b*, and *Otsclock1b *clustered together and orthologs *OmyClock1a *and *Otsclock1a *clustered together in both NJ and MP trees (Figure 2A and 2B). Thus, the orthologs within the salmonids were more similar than paralogs (e.g. *OmyClock1a *clustered with *Otsclock1a*, not *OmyClock1b*).

**Figure 3:**
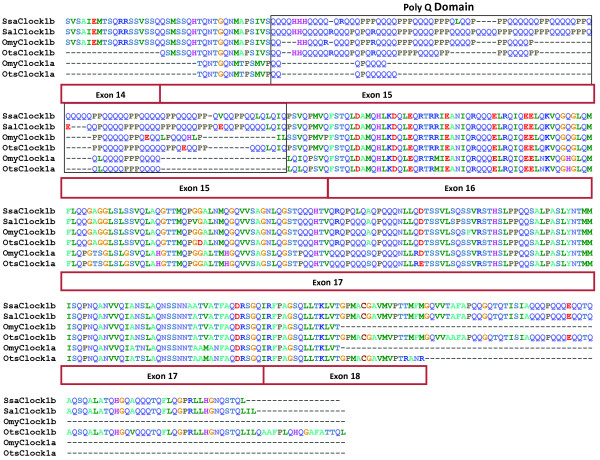
**Protein alignment of SSACLOCK1B, SALCLOCK1B, OMYCLOCK1B, OTSCLOCK1B, OMYCLOCK1A, and OTSCLOCK1A demonstrating the variable polyglutamine (PolyQ) repeat region (black box)**. Exons are indicated by the burgundy boxes.

Protein alignments of the salmonid CLOCK1 sequences revealed that a polyglutamine motif region (PolyQ) in exon 15 was present in all the orthologs and that this region varied in length between duplicates and across the species (Figure 3). SALCLOCK1B and SSACLOCK1B contained the highest number of glutamine residues with a total of 63 in this region. OMYCLOCK1A contained 18 glutamine residues, whereas OMYCLOCK1B contained 36 glutamine residues. Similar to what was found for rainbow trout, Chinook salmon OTSCLOCK1A and OTSCLOCK1B contained 20 and 41 glutamine residues, respectively [27]. Other taxa show differences in the CLOCK PolyQ regions between species and even within a species. For example, this region varies in *Drosophila *species with glutamine counts ranging from 25 to 33 residues [34] and in avian species, like blue tits (*Cyanistes caeruleus*) and bluethroats (*Luscinia svecica*), where glutamine counts vary from 9 to 17 and 10 to 16, respectively [35].

Some species exhibit variable PolyQ tract lengths according to latitudinal gradients. For example, within species length variants were observed in blue tit and Chinook salmon [35,36]. Most importantly, these PolyQ length and latitudinal gradients appear to correlate with breeding/spawning times. At higher latitudes, mean PolyQ lengths were longer compared to lower latitudes [35,36]. Differences in PolyQ lengths also occurred in Chinook salmon that spawn at temporally distinct times of year in the same river [9]. Interestingly, no variation was found in the PolyQ region of OTSCLOCK1A [36]. In this current study, the presence of variable PolyQ regions in CLOCK1B across the salmonids suggests that this region may be a result of local adaptation to latitude affected ecological factors like photoperiod.

The presence of variable PolyQ regions in CLOCK1B across the salmonids suggests that this region may be important in altering circadian phenotype. PolyQ lengths have been associated to fitness parameters in other species. In a single blue tit population, Liedvogel et al. [37] found evidence that short CLOCK PolyQ regions were correlated with earlier breeding times for females and shorter incubation periods for both males and females. In terms of fitness, females that had shorter PolyQ regions had a higher number of fledged offspring [37]. Thus, Chinook salmon populations inhabiting more northern latitudes may delay reproductive timing in relation to seasonal photoperiodicity in order to enter more favourable stream environmental conditions for spawning. However, direct comparisons of spawn timing events in female salmonids with different PolyQ tracts still need to be examined to test this hypothesis.

Acute effects of PolyQ and 3' Q-rich modulation on circadian rhythms have also been observed. Deletion of 51 amino acids in the 3' Q-rich region in mutant mice has been associated with lengthening the circadian period up to 4 hours in homozygous mice and eventual loss of rhythmicity over a few weeks in darkness [38-40]. Thus, PolyQ and 3' Q-rich variation appear to be important component of transcription activation [41-43]. Since the PolyQ domain is involved in transactivation, the domain size is thought to directly affect the transcription of the target genes and thus phenotype [42].

### Mapping of Clock and amh

#### Homologies and homeologies

**Figure 4:**
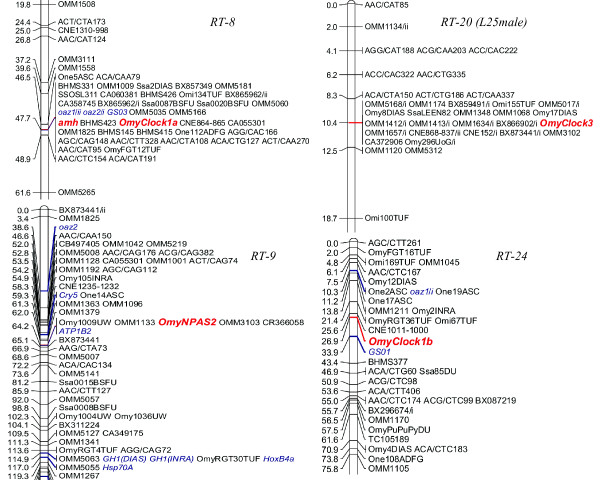
**Genetic map positions for *Clock1a*, *Clock1b*, *Clock3*, *NPAS2*, and *amh *in rainbow trout mapping panels**. Unless identified, the linkage groups were from merged family maps of the female mapping parents. Additional mapping locations of known gene markers are shown in blue italic font.

**Figure 5:**
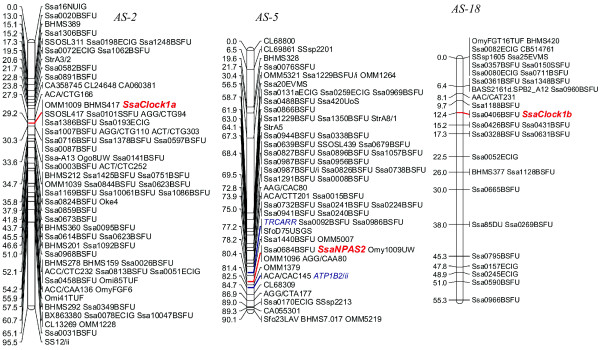
**Genetic map positions for *Clock1a*, *Clock1b*, *Clock3*, and *NPAS2 *in Atlantic salmon mapping panels**. The linkage groups were from merged family maps of the female mapping parents. Additional mapping locations of known gene markers are shown in blue italic font.

**Figure 6:**
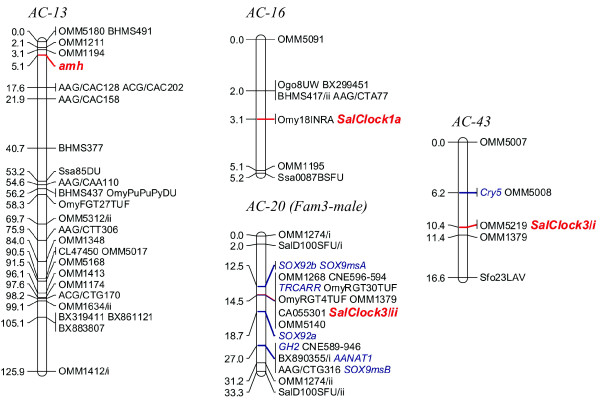
**Genetic map positions for *Clock1a*, *Clock1b*, *Clock3*, and *amh *in Arctic charr mapping panels**. Unless identified, the linkage groups were from merged family maps of the female mapping parent. Additional mapping locations of known gene markers are shown in blue italic font.

In rainbow trout, *Omyclock1a *mapped to the central region of linkage group RT-8 while *OmyClock1b *mapped to the short arm of (i.e., RT-24p) (Figure 4). *amh *mapped to linkage group RT-8 supporting previous findings [28], while its putative paralogous copy was localized to linkage group AC-13 in Arctic charr, which is homologous to RT-24p arm in rainbow trout (Figures 5 &6). The mapping of Clock1 duplicates in rainbow trout and putative amh paralog assignments provides further evidence for homeologous affinities of RT-8 and RT-24 [25,44]. Additional duplicated genes have been mapped to these linkage groups including ornithine decarboxylase antizymes (*oaz1/i/ii*) [45] and glutamine synthetases (*GS01/GS03*) [46] (Figure 4). Although RT-24 and RT-8 are the fourth and fifth largest chromosomes in rainbow trout, respectively, the homeologous affinities of these linkage groups are largely unknown [47]. The long arm of RT-24 shows homeologous affinities to the long arm of RT-23 based upon a single duplicated SSR marker assignment (Omy27INRA) [48] as well as a duplicated conserved non-coding element (CNE1056-1058) [26].

The mapping of the *OmyNPAS2 *gene to the centromeric region of RT-9 (i.e., RT-9p arm) also supports some intriguing evolutionary affinities between the RT-8/9/24 chromosomal regions in rainbow trout (Figure 4). Centromeric regions of RT-24 and RT-9 share a degree of putative homeology based upon the syntenic mapping of a pair of duplicated markers (Omy1231INRA and Omy1287INRA) [49]. Similarly, homeology between the short arms of RT-8 and RT-9 also exists based upon the mapping of the duplicated marker OMM1825 [49], a duplicated CNE (CNE1232-1235) [26], and duplicated copies of the *oaz2 *genes [45]. However, the homeologous segments between RT-8/9 appear to be more telomeric in location on the RT-9p arm compared to those between RT-9/24.

A single copy of the *OmyClock3/NPAS2-like *gene was mapped to the central cluster of markers on the RT-20 linkage group in a male rainbow trout (Figure 4). RT-20 shares homology with both arms of linkage group AS-28 and given that AS-19/28 are homeologous, this region may also possess homology to linkage group arm AS-19q [25]. For the RT-20 linkage group, homeologies are only characterized extensively for the 14p/20q arm duplications, although a pair of duplicated markers also indicates that RT-14q may share a degree of homeology to RT-25q. There is also evidence that a small region of homeology may exist around the centromeric regions of both RT-20 and RT-9 based upon the presence of up to 3 duplicated markers that have been genotyped in different mapping panels [25,50].

In Atlantic salmon, *SsaClock1a *and *SsaClock1b *mapped to the AS-2qa region of AS-2 [51] and AS-18, respectively (Figure 5). AS-18 represents an acrocentric chromosome in Atlantic salmon, which shares homology to only a single rainbow trout linkage group arm (i.e., RT-24p), and thus is not designated with respect to arm regions in the species. AS-2, a fused acrocentric chromosome in Atlantic salmon, shares homology to two rainbow trout linkage group arms (i.e., RT-8q in the AS-2qa region proximal to the centromere, and RT-27q in the more distal AS-2qb region [51]). *SsaNPAS2 *mapped to AS-5, and more specifically to the AS-5qb region of the linkage group. AS-5 is also a fused acrocentric chromosome in Atlantic salmon, with the AS-5qa region homologous to RT-22q, and the AS-5qb region homologous to RT-9p [51]. Interestingly, while the *Clock1 *duplicates in rainbow trout, along with *NPAS2 *gene copy appear to fall into regions that may possess a degree of ancestral homeology (based upon the syntenic mapping of additional duplicated genetic markers), the observed mapping locations for these genes in Atlantic salmon is not supported by any additional duplicated marker information (however, see below for discussion on ancestral homologies).

In Arctic charr, *SalClock1a *and *SalClock1b *were localized to linkage groups AC-16 and AC-13, respectively, and *SalClock3/NPAS2-like *duplicates mapped to AC-20 and AC-43 (Figure 6). Similar to Atlantic salmon, there are no known homeologies recognized between AC-13/16, or AC-20/43. Current mapping data indicate that segments of AC-13 may share homeology to AC-14 (1 marker) and AC-34 (9 markers), while a single duplicated marker supports potential homeology between AC-16 and AC-27 [[52]; unpublished data]. AC-43 does not share any duplicated markers with other linkage groups in the Arctic charr genome, while AC-20, appears to be a fused metacentric chromosome possessing chromosome arms that are homeologs to one another [52]. These linkage group arms are homologous to the RT-2q and RT-9q linkage group arms in the rainbow trout genome [25].

Homologous linkage group alignments between the species indicate that AC-16 is homologous to the RT-8 linkage group. Given the tight clustering of markers on RT-8 due to the lack of recombination in the female mapping parents used to construct the rainbow trout genetic linkage maps [53], the actual RT-8 arm assignments to AC-16 are difficult to determine. However, within the cluster of markers on AC-16, two markers (Ogo8UW and BX299451) are evident, and these are located quite distally along the RT-8q chromosome arm (see Figure 4). This would suggest that this rainbow trout chromosome arm (i.e., RT-8q) shares homology to AC-16, and this interpretation is also compatible to the assignment of shared homology to the AS-2qa linkage group arm.

The assessment of homology for the *SalClock1b *mapping location on AC-13 is even more difficult given the fact that the linkage assignment is only based upon male segregation data (See Figure 6). AC-13 appears to be a metacentric chromosome in Arctic charr with the AC-13p arm homologous to the RT-24p arm, and the AC-13q arm homologous with RT-14p or RT-20q linkage group arms [25].

Since the mapping location of *amh *in Arctic charr is clearly homologous to the RT-24p arm (Figures 4 and 6) in rainbow trout, and given the syntenic clustering of *Clock1 *and *amh *on RT-8, it may be inferred that the homology of the *SalClock1b *copy is to the RT-24p arm. The differential localization of *amh *copies to RT-8 and AC-13 indicates that duplicates of this gene may still exist in both rainbow trout and Arctic charr on the RT-24p, and AC-16 linkage groups, respectively. Alternatively, following the 4R duplication of these genes, one of the two duplicated copies may have been differentially silenced within the respective alternate homeologous linkage group arms of either species.

Mapping of the duplicated *SalClock3/NPAS2-like *copies to AC-20/43 is particularly intriguing, given the fact that these linkage group regions have distinct homologies to separate linkage group arms in rainbow trout and Atlantic salmon. AC-43 is homologous to the RT-9p arm (Figures 4 and 6), and the AS-5qb (Figures 5 and 6) linkage group arms. Both of these linkage group regions possess a copy of the *NPAS2 *gene, as previously mentioned. Given that a more precise localization of *OmyClock3/NPAS2-like *is not possible based upon the male genotyping data obtained in this study, it is reasonable to assume that this gene maps to the RT-9p arm as well, given that AC-43 and RT-9p share homology. If, however, we consider that the duplicated copy of the *SalClock3/NPAS2-like *gene maps to AC-20 (which share homology with only the RT-9q arm) then two copies of these genes may in fact exist on the RT-9 linkage group in rainbow trout (see below).

#### Affinities to ancestral Actinopterygian linkage groups

Comparative synteny analyses between the salmonids and model fish species, such as zebrafish, stickleback and medaka, can be used to determine the origin of ancient genomic regions in fish species. These comparative genomic studies suggest that modern day ray-finned fishes descended from an ancestral vertebrate lineage that had 12-13 linkage groups (A-M) [54-56]. Using information on the salmonid-specific linkage group arm homologies to the ancient ancestral linkage group arm origins [25], the duplicated *Clock1 *and *amh *copies in the salmonid species have likely arisen from the M ancestral lineage. Linkage group arms RT-8q; RT-24p; AS-2qa and AS-18 all share homology to the M ancestral lineage.

BLASTN alignments of the *OmyClock1b *gene to the medaka genome also indicate that there may be an unannotated *Clock *gene copy within Ol-4 at 1.755 Mb. The Fgenesh-M analysis [57] suggests that this assignment may be a pseudogene signature given that the alignment blocks do not overlap any of the predicted exons within the region. Regardless, this region would be considered most similar to *OmyClock1b*, making Ol-4 the most informative syntenic block for ancestral linkage group assignments. Using this information, the association of Ol-4 to ancestral lineage M is compatible with the major ancestral assignments in the salmonids [25]. Ol-4 genes also share a synteny block with the *Clock *copy on Dr-20 (spanning from the *Clock *gene at 21.381 Mb-23.259 Mb, and homologous to the region from 0-1.733 Mb in medaka), suggesting homology to the M lineage. The *Clock *copy in sticklebacks (0.489 Mb on Ga-IX), however, shares greatest homology to a region on Dr-1 from 13.624-26.952 Mb, and to Ol-1 within the region from 14.996-15.785 Mb on this medaka chromosome. *Clock3 *in zebrafish is localized to Dr-1 at 18.849 Mb, while *Clock3 *in medaka is localized to Ol-1 at 14.996 Mb. Kasahara et al. [55] indicate that Ol-1 is derived from a mosaic mixture of genes that derive from the E and F ancestral lineages in fish indicating that *Clock3 *gene copies likely have a different teleost-specific ancestral chromosomal origin compared to the *Clock1 *copies. This would then suggest that the region of Ga-IX possessing *Clock *derives from a different origin than the Clock copies on Dr-20 and Ol-4, and is in fact more similar to *Clock3 *copies in zebrafish and medaka.

The single copy of *OmyClock3 *mapped to RT-20 in rainbow trout is compatible with a hypothesis that this *Clock3 *gene arose from the F ancestral lineage as genetic markers possessing homology to both Ol-1/-18 are located on the RT-20q linkage group arms [25]. However, the copy of *SalClock3/NPAS2-like *that is located on AC-20, would suggest that homology to the RT-9 linkage group may exist for two Clock gene copies. AC-43 is homologous to RT-9p (*SalClock3/NPAS2-like/i *location) while AC-20 (*SalClock3/NPAS2-like/ii*) is homologous RT-9q. The copy on AC-43 is likely *NPAS2*, as this matches the location of this gene in rainbow trout. The copy on AC-20, may, however, be representative of *Clock3*. While it is true that there is extensive homology to the E ancestral grouping on the RT-9q linkage group arm, there is also a small region of homeology to the G lineage around the centromere representative of duplicated markers between RT-9/20 (as previously mentioned).

In stickleback, the most likely *OmyNPAS2 *BLASTN homology was to a pair of partial exons at 17.213 Mb on Ga-VII while the *NPAS2 *gene has been assigned to chromosome Ga-VII at 17.209 Mb. Interestingly, both Ga-VII and Ga-IX appear to share extensive homeology to one another throughout the proximal regions of both linkage groups. The region designated as possessing *Clock *on Ga-IX is not homologous to the ancestral M lineage regions containing the salmonid Clock1a and Clock1b copies. These regions are, however, homologous with the chromosome segments of Ol-18 and Dr-7 within Ga-VII, and with Ol-1 and Dr-1 within Ga-IX, which clearly indicates their affinities to the F ancestral lineage of teleost fishes [25,55,56]. This would indicate that the "Clock-like" copy designated as *NPAS2 *on Ga-VII within the ENSEMBL 56 release is, in fact, a duplicate copy of *Clock3*, with the homeolog of this gene still evident on Ga-IX.

BLASTN homologies of *OmyNPAS2 *within the medaka genome are to chromosome Ol-14 at 17.143 Mb, and to Dr-5 at 20.844 Mb in zebrafish and both of these regions are descended from the GH ancestral lineage [25]. The Ol-14 region possessing *NPAS2 *has extensive homology to Dr-21, while the Dr-5 region containing *NPAS2 *has extensive homology to Ol-10 and -14. To highlight the similarity in these gene copies, the first exon region (1-155 bp) of the *OmyNPAS2 *gene also has extensive homology to the zebrafish *Clock3 *gene on Dr-1 (85.1% identity within 148 bp), and *Clock1 *copy on Dr20 (82.1% identity within 162 bp). In addition, the *OmyNPAS2 *sequence has a high degree of homology to an unannotated sequence block (85.8% identity for 148 bp) within Dr-15 between the genes *LOC568103 *and *kitb*. Both *kita *and *kitb *genes are companion syntenic genes to the *Clock *genes among vertebrates. The copy on Dr-15 may be a functional *NPAS2 *copy based upon the Fgenesh-M analysis [57] that is currently unannotated, and given the fact that major chromosomal segments of Dr-10, -14, -15, and -21 are derived from the GH lineage, it would suggest that the BLASTN alignment of the *OmyNPAS2 *gene on Dr-15 represents a 3R homeolog of *NPAS2*.

In summary, it can be stated that the *Clock1 *homeologs in rainbow trout and Atlantic salmon are all derived from ancestral linkage group M. The genomic regions surrounding *NPAS2 *are derived from ancestral linkage group GH, and regions surrounding *Clock3 *are derived from teleost ancestral linkage group F. At present, it is not possible to clearly assign the *OmyClock3/NPAS2-like *copy on RT-20 to an origin either from the GH or F lineages because homeologies exist both between RT-9/20 (possible GH lineage) and RT-14/20 (possible F lineage).

By tracing ancient linkage groups GH and F even further back in vertebrate evolution it is possible to ascribe the origins of the proto-Actinopterygian linkage groups to 10-13 putative proto-vertebrate linkage groups (designated A' - J' or possibly A' - M') [56]. These linkage groups underwent two rounds of whole genome duplications and extensive fusions prior to a further 3R duplication in the teleost fishes. When these lineages are configured within the 12-13 proto-Actinopterygian linkage groups it is intriguing to note that almost all of ancestral linkage groups G and F are derived from C' lineage blocks (i.e., C1 and C2) [56]. Thus, the extensive homology observed between the *NPAS2 *and *Clock3 *gene copies across the various teleost species compared may not be that surprising given that they are likely derived from multiple gene duplications from the same ancestral gene. Additionally, two lineages of *NPAS2 *genes exist in vertebrates (a teleost-specific form and Sarcopterygian form) with the teleost-specific form more closely related to *Clock3 *and *Clock1 *vertebrate genes [see data for family TF324568 in: http://www.treefam.org]. This suggests differential silencing and retention of paralogous copies of the *NPAS2 *gene following either the 1R or 2R duplication events in the vertebrate lineages. The extensive overlap of inferred homeologies seen within rainbow trout in the current study for the *NPAS2 *and *Clock3 *copies is also consistent with these observed phylogenetic affinities.

#### Co-localization of Clock and amh with life-history and growth QTL

In this current study, *OmyClock1a *and *amh *were localized in the central cluster of markers with zero recombination on RT-8 including OmyFGT12TUF, OMM1009, and SSOSL311, while *OmyClock1b *and *amh *(Arctic charr maps) were mapped in close proximity to BHMS377 and OmyRGT36TUF markers (Figure 4), which are homologous to RT-24 in rainbow trout. These markers fall within genomic regions that contain QTL for important life-history traits in salmonids such as spawning time, early maturation, and development rate [8,44,58-64] suggesting that *Clock1 *homeologs and putative *amh *duplicates may be candidate genes affecting these traits.

The strong spawning time QTL located on RT-8 in a backcross family (Lot44) [58] accounted for up to 50-60% of the trait variation [8,58,60]. Due to the lack of recombination that has been detected in both sexes within this chromosomal region [47], it has been difficult to assess the chromosomal arm location for these QTL effects. However, using the comparative synteny mapping approaches outlined above, it appears that the most likely locations for these genes fall within the RT-8q arm (i.e., derived from the M ancestral lineage). Counter to this observation, the strongest early maturation QTL region on RT-8 was localized to the marker OMM1304 on the RT-8p arm [44], indicating an origin to the I ancestral lineage of teleost fishes [25]. However, relatively strong QTL for this trait were also detected for several makers falling within the central cluster of markers which could, therefore, indicate interaction effects among these regions.

Development rate QTLs have also been localized to the central region of RT-8. For example, Sundin et al. [61] suggested that a development rate QTL was located on this linkage group (associated with markers OmyFGT12TUF, OMM1009). A subsequent study, using doubled haploid lines of rainbow trout confirmed that a major developmental rate QTL was localized in the central clusters of RT-8. This QTL explained a large proportion of the phenotypic variation (23.2%) associated with the trait [64].

*OmyClock1b *also mapped to a region of the rainbow trout genome (i.e., RT-24) with strong influences on variation in life-history traits. Regions on RT-24p (BHMS377 and OmyRGT36TUF) occupied by *Clock1b *explained up to 20% of variation in the spawning time of females [8,60]. Maturation timing QTL have also been localized to RT-24 [44,59] but given the fact that these QTL were localized in male parents, the determination of the chromosome arm affinities was not possible. Similarly, a small effect development rate QTL (one of four along with RT-9, -10, -14) was localized to RT-24, which explained approximately 1.26% of phenotypic variation [64].

Other locations of Clock-like genes are also associated with life-history QTL in rainbow trout. The *NPAS2 *copy mapped on RT-9 may correspond to one of the developmental rate QTL identified in the Nichols et al. [64] study. Additionally, a strong QTL for the timing of smoltification in rainbow trout has been localized to RT-20 [65] which may be related to *Clock3 *functional differences.

In Arctic charr, *SalClock1a *was localized to AC-16, and *SalClock1b *and *amh *were localized to AC-13 both of which contain suggestive female maturation time QTL. On AC-16, *SalClock1a *is closely linked (0 cM recombination) to Omy18INRA and BHMS417, markers that suggestively influence age at sexual maturation in females [63]. Likewise, *SalClock1b *and *amh *were linked to OMM1211 on AC-13 in the Arctic charr maps at 0 cM and 3 cM recombination, respectively. This linkage group contains a suggestive age at maturation QTL centered on OMM1211. Both QTL on AC-16 and AC-13 explained approximately 7% of the phenotypic variation [63].

Studies investigating genetic associations with life-history variation in Atlantic salmon have not been as extensive as those with rainbow trout or Arctic charr. Fotherby [62] identified 11 putative QTL for early maturation in female Atlantic salmon (at 3 years), but only two of these (i.e., AS-17 and AS-28) overlapped with a region homologous to a rainbow trout linkage group containing a possible Clock-related gene effect. The region identified on AS-17 (marked by One114ADFG) is homologous to the RT-8p arm. One114ADFG is in fact only 4 cM distal to OMM1304 in female rainbow trout genetic maps, where the strong maturation timing QTL was detected in the Haidle et al. [44] study. A marker located distally on AS-28, BX873441, has homology to the distal region of RT-9p, while a proximal marker, CL47450, shows homology to rainbow trout duplicated homeologs RT-14/20. As mentioned above, RT-9p possesses an *NPAS2 *copy, while *Clock3 *maps to RT-20, and may possess a duplicate within either the RT-9/20 or RT-14/20 homeology groups.

Rainbow trout is perhaps the most characterized salmonid species with respect to growth QTL. QTL on RT-8, RT-9 and RT-24, which also coincide with the location of Clock-related genes, have strong influences on body weight (BW) [66]. While significant BW QTL were also identified on RT-14 and RT-20, these linkage groups were not observed to be significant across all the parents tested, and thus were not considered to house major BW species-specific QTL regions. Future studies across multiple families in different strains may lead to a different conclusion. The association among variation in multiple life-history and phenotypic traits with Clock-related chromosomal regions suggests the existence of conserved blocks of genes regulating single or multiple traits (discussed in the next section) [44,61].

### Synteny analyses: conserved blocks of reproductive and cell cycling genes

Synteny analysis across teleost species revealed a conserved block of genes surrounding the *amh *gene which suggests the presence of a functional cluster [67]. Comparative analysis of a*mh *containing regions on medaka chromosome 4, tetraodon chromosome 1, and zebrafish chromosome 22 revealed conserved synteny of 12 genes, including *lhx7*, *hsd11b*, *map2k2*, *LINGO3*, *oaz1*, *DOT1L*, *ELL*, *vtg*, *ddx59*, *nr5a2*, *kif14*, and *lhx9 *involved with sexual maturation and cell cycling. With the exception of *DOT1L*, all these genes appear to exhibit circadian oscillations in one or more mammalian tissues, suggesting either direct, or CCG-mediated regulation via *Clock *gene expression [68] (Additional File 2). When the teleost genomic regions surrounding *amh *were compared to the human chromosome 19, there was conserved synteny of *DOT1L*, *oaz1*, *LINGO3*, *map2k2*, and *hsd11b *genes. *Clock *was found on the same chromosomes as *amh *within medaka and tetraodon, and *Clock *was tightly linked to *kita *across all the species investigated (Additional File 2).

Genes regulating reproduction include *lhx9*, *lhx7*, *nr5a2*, *hsd11b3*, and *kit*. For instance, members of the LIM homeobox (*lhx9 *and *lhx7*) are found adjacent to *amh *in medaka, tetraodon, and zebrafish. *Lhx9 *is essential for gonadal development in mice as *Lhx9*-deficient mice developed without gonadal tissues [69,70]. *Lhx7 *(or *lhx8*) code for a transcription factor that plays a critical role in folliculogenesis. An *Lhx7 *deficiency results in altered expression of oocyte-specific genes in the ovary, and is therefore thought to be critical in follicle formation and differentiation and maintenance of oocytes [71]. Likewise, *LRH-1 *(*nr5a2*) is found upstream to *amh *in the teleost species. This gene is an orphan nuclear receptor, is a critical regulator of multiple mechanisms essential for maturation of ovarian follicles and for ovulation [72]. *LRH-1 *mutants resulted in increased estradiol synthesis and downregulation of genes (*Scarb1*, *Star*, *Cyp11a1*) critical for luteinization [72]. Because of these effects, it is thought that *LRH-1 *is primarily important for female sexual function relative to *SF-1 *(*nr5a1*), which is involved in male sexual development [73].

As well, the *Hsd11b3 *gene is found adjacent to *amh *in medaka, tetraodon, zebrafish and fugu (Additional File 2). HSD11B3 (11β-hydroxysteroid dehydrogenase type 3) is a steroidogenic enzyme that is involved in the production of 11-ketotestosterone [74] the main androgen sex steroid which effects spermatogenesis and secondary sexual characteristics in male teleosts [75,76]. Mutations within the *Hsd11b3 *gene have also been linked to pseudohermaphrodism in human males due to altered sex steroid ratios [77]. In both sexes, HSD11B3 is also important for the conversion of glucocorticoids into inert forms [78]. Corticosteroids have been linked to suppression of steroidogenesis in mammals, therefore HSD11B enzymes are thought to be important in protecting the developing gametes from high cortisol levels [79-81].

In addition to its functions related to melanogenesis and haemopoiesis the *c-Kit *gene is also important in regulating gametogenesis in vertebrates [82], and has also been implicated in lipid metabolism as mice deficient for this gene exhibit steatosis of the liver during development [83]. *Kit *is found adjacent to *Clock *and *amh *in medaka, and tetraodon while in zebrafish, fugu, human, and mouse *Kit *and *Clock *are adjacent on different chromosomes than *amh*. Kit tyrosine-kinase receptor and its ligand (KL) are essential for spermatogenesis as they upregulate genes that promote mitosis, and meiosis entry of spermatogonia [84]. Also, *Kit *is involved in oogenesis and follliculogenesis in female mammals (reviewed in [85]). Direct Clock-related regulation of this gene may also occur through retinoic acid stimulation of Activator Protein 2 (AP-2) expression. AP-2 as well as bHLH binding sites are present in the *c-Kit *gene [86], and Shirai et al. [87] have shown than there is a bidirectional regulation of *Clock*/*bmal1 *regulated genes and retinoic acid levels mediated through the expression of retinoic acid orphan receptors.

Specific to the fishes, vitellogenin (*vtg*) is found downstream to *amh *(Additional File 2). Vitellogenin is a precursor yolk protein in fish eggs, and it gene is regulated as a result of estrogen binding [88]. Estrogen levels and indeed sex steroid production in general may be directly influenced by CLOCK levels, as the functional expression for one of the key enzymes regulating their production (i.e., steroidogenic acute regulatory protein or StAR) has been shown to be altered via *Clock*/*bmal1 *expression [89]. Thus *Clock *expression may provide direct regulation of subsequent VTG production.

Direct regulation of AMH production via *Clock *gene expression is also considered possible given the fact that Steroidogenic Factor 1 (SF-1) binding sites (a downstream CCG gene) have been identified within the 5' region of the *amh *gene in both mammals [90] and fish [91]. *SF-1 *(*nr5a1*) is the closest nuclear orphan receptor family member to the *LRH-1 *gene (*nr5a2*) [92]. Given the more prominent role of LRH-1 in ovarian development, compared to SF-1 [73] it would be of interest to investigate potential cis-regulation of *amh *via *nr5a2 *in future studies. Given the interactions that several of the '*Clock*/*amh*-syntenic' genes exhibit in the formation and maintenance of gonadal structures in both male and female teleosts, we may consider that the genes found in this cluster represent a 'reproduction controlling block'.

Genes involved in cell cycling (*kif14*, *map2k2*, *DOT1L*, and *oaz1*) are also found in conserved synteny surrounding *amh *across the species investigated. For example, *Kif14 *is found upstream to *amh *in medaka, tetraodon, and zebrafish (Additional File 2). *Kif14 *codes for a kinesin motor protein that regulates intracellular movements. Silencing of KIF14 using strong RNA interference resulted in cytokinesis failure while weak RNA interference-mediated silencing caused apoptosis of cells during various stages of mitosis [93]. Likewise, *Map2k2 *is found tightly linked with *amh *across the vertebrate species examined. MAP2K2 is part of a signal transduction pathway (MEK/ERK/MAP kinase) that promotes cell cycling. Similarly, *DOT1L *is tightly linked to *amh *among teleosts, and this gene codes for a histone methyltransferase (H3K79) that is associated with meiotic checkpoint control, and DNA damage response in yeast [94,95]. DOT1L deficient mice demonstrated developmental abnormalities that ultimately lead to death [96]. *oaz1*, like *DOT1L *and *map2k2*, is tightly linked to *amh*, and this gene codes for a protein that binds to and promotes degradation of ornithine decarboxylase (*ODC*), a protein that is involved in the regulation of mitotic turnover rates by controlling polyamine levels [97-99].

Interestingly, some of the genes in this conserved cluster are involved in transcription control, cell cycling, and regulation of circadian rhythms. For example, both *ELL *and *ddx59 *expression is associated with RNA metabolism and these genes are adjacent to *amh *(Additional File 2). DDX59 is a member of the DEAD-box proteins, which are intimately involved in RNA metabolism as they regulate translation and gene expression in organelles (reviewed in [100]). ELL functions as an RNA polymerase II elongation factor that increases the overall rate of transcription, and regulates mitotic phase transitions during the cell cycle [101]. ELL also regulates glucocorticoid steroid receptor responses, which may partially modulate CLOCK:BMAL1 heterodimer activation [102], but does not influence androgen and progesterone receptors [101].

There is evidence that gene order on chromosomes is not random in zebrafish, and in fact, co-expressed genes tend to be found in close proximity to each other [103] comprising functional clusters. Overall, the comparative genomic analysis among the teleost species shows that 'Clock-containing' chromosomal segments are highly conserved. We would therefore predict that the genes found in this syntenic cluster will also co-occur on RT-8 or its homeolog RT-24 (i.e., two of the strongest life-history QTL regions in rainbow trout). However, additional life-history QTL regions may also be partially regulated via *Clock *expression if they possess CCGs.

### Clock mediated regulation of reproductive/cell cycling genes associated with salmonid life-history QTL regions

In mammalian systems, microarray studies of circadian gene expression indicate that most circadian regulated genes are either directly modulated via Clock or Clock-inducible gene expression as the majority of genes identified as showing circadian rhythms in their expression possessed E-box, AP-2, CRE, SP1 and EGR transcription factor binding sites in their 5' regulatory regions [68]. CLOCK:BMAL1 heterodimers bind to E-box sequences in the 5' regions of genes and directly influence their expression. Such CCGs are therefore considered to be under direct circadian control. Some of these genes are implicated in reproduction and growth and have been mapped to rainbow trout life-history QTL regions, or may reside within such QTL regions based upon putative homologous synteny comparisons among teleost species [25]. For example, both Gonadotropin (*GnRH*) and gonadotropin receptor (*GnRHR*) promoter regions contain several non-canonical consensus E-boxes (CANNTK) sites [104,105]. *GnRH *and *GnRHR *expression patterns were disrupted by the *Clock *mutation in mice [6,105]. As a result, *Clock *mutant mice exhibited prolonged estrous cycles and were subfertile [6]. *GnRH *maps to RT-30 and RT-6 [8] and the copy on RT-30 may contribute to a strong maturation timing QTL in rainbow trout [44].

Another gene potentially coupled to QTL variation in rainbow trout is estrogen receptor beta (*ERβ*, *nr3a2*), which is a member of the nuclear receptor superfamily and a key mediator of estrogen action [106,107]. *ERβ *shows circadian oscillations in expression and not surprisingly has an E-box element in the promoter region which allows for direct *Clock *gene control [108]. A copy of *ERβ *(*esr2a*) is located less than 1 Mb away from the *Clock *gene on zebrafish chromosome Dr-20, which would make this gene a contributing candidate to the DR, SPT and EM QTL that have been detected on RT-24. Another gene that is related to reproduction and is under direct *Clock *gene control is *StAR*, which regulates the rate-limiting step of steroidogenesis. StAR showed circadian rhythms, and contains E-box regions in promoter regions that allow CLOCK:BMAL1 to activate transcription [89]. *bmal1 *knock-out mice were infertile and gonadotropin levels were altered [109]. Alvarez et al. [109] found that *StAR *expression was not altered in *Clock *mutant mice; however, they did not assess the effects of the Clock homolog *NPAS2 *mutants. *StAR *maps to 45.651 Mb on Dr-8, and to 18.480 Mb on medaka linkage group Ol-9 which makes this gene part of the I ancestral lineage of teleost fishes [55]. Using comparative synteny blocks to the rainbow trout genome, it is inferred that the most likely map position for this gene with either be the RT-10p or RT-19q linkage group arms [25]. EM and SPT QTL have been localized to RT-19 [8,44].

Some of the cell-cycle genes (e.g. *Wee1*) involved in either G2-M or G1-S transitions contain E-boxes [110] indicating their status as a CCG. Wee1 is a protein kinase known to be involved in mitotic entry checkpoint in eukaryotic cells as it regulates phosphorylation status of CDC2 whose activity is required for a cell to enter mitosis. Besides growth, cancer is related to cell-cycling, and as such, CLOCK:BMAL1 heterodimers regulate the expression of *c-MYC *oncoprotein, which cooperates with *ATM *(ataxia-telangeictasia mutated), in promoting apoptosis and suppressing tumorigenesis [111]. NPAS2:BMAL1 heterodimers coincidentally influence *c-MYC *expression [112]. A recent PCR array study demonstrated that NPAS2 knockdowns *in vitro *resulted in significant effects on genes involved in cell cycling and DNA repair, which further supports that circadian genes may play a role in tumorigenesis and cell cycling [111,113]. Rainbow trout linkage group regions with greatest homology to Dr-18 and Ol-6 where *Wee-1 *has been mapped are the homeologous pair RT-10q/18, and may be associated with a moderate DR QTL effect on RT-10 [64].

Several genes influencing reproduction and growth contain potential *Clock/bmal1 *regulatory sites (E-box sequences) and demonstrate circadian oscillations in certain tissues within the mouse [68]. For example, promoter regions of follicle stimulating hormone receptor (*FSHR*), androgen receptor (*AR*) and growth differentiation factor 9 (*Gdf9*) contain E-box sequences in their promoter regions, and thus may be partially regulated in a CCG fashion [114,115]. As hormone receptors, both FSHR and AR expression is required to enable the signal transduction of FSH and testosterone, in order for spermatogenesis to occur [116]. GDF9 is critical for normal ovarian function as it regulates key genes (i.e. kit ligand, LH receptor, prostaglandins, and progesterone) involved in folliculogenesis [115]. As well, *SF-1 *(*nr5a1*), an orphan nuclear receptor that regulates the expression of the steroidogenic genes and sexual differentiation (reviewed in [73]) contains E-box sequence in the promoter region [117]. Luteinizing hormone itself also appears to be under circadian control as the LH surge required in proestrous was not seen in *Clock *mutant mice [118]. *LH *itself contains E-box motifs in the promoter region [119], which suggests that LH production is under direct CLOCK:BMAL1 control. Upstream control may also occur with vasopressin, an LH regulator, which is directly regulated by CLOCK:BMAL1 [118].

Currently mapping data is only available for *SF-1 *in zebrafish, making syntenic comparisons to the rainbow trout genome difficult. For the other 4 genes, however, it is known that *LH *maps to RT-17 (i.e., *GTH2B*) [120], and it is also predicted that *FSHR *maps to RT-17q as this gene is located on Dr-12 at 29.005 Mb, and on Ol-19 at 11.455 Mb. This region is reported to possess a strong EM QTL [44]. Interestingly, both *AR *and *Gdf9 *may also map to a single genomic region (i.e., the homeologous pair RT-3q/25p), as *AR *maps to Dr-5 at 35.987 Mb, and within Ol-10 at 18.354 Mb, while *Gdf9 *maps to Dr-14 at 23.776 Mb and Ol-10 at 13.566 Mb, making both these genes likely descendants from the GH Actinopterygian chromosome lineage [25]. Moderately strong EM and SPT QTL have been localized to RT-3 [8,44].

## Conclusions

Multiple Clock-family gene copies have been identified in this study (i.e., up to 5 in rainbow trout), that map to duplicated homeologous linkage group positions in the various salmonid species investigated. The NPAS2 and Clock3 genes within this family appear ancestrally more closely related, than do the Clock1 copies, and the physical linkage of the Clock1 gene duplicates to major life-history QTL regions in salmonid fishes, posits their functional role in regulating many of the associated life-history traits that have been studied in these fishes. Intriguingly, many genes important in regulating growth and life-history events are known to occur in sytenic clusters close to the Clock gene clusters. As an example, one of the most important genes regulating maturation timing in salmonids (i.e., anti-mullerian hormone), was mapped within a syntenic region of the Clock1a gene in rainbow trout. However, in Arctic charr, this gene was located within the Clock1b gene syntenic block, indicating differential silencing of the duplicated copies following the salmonid specific 4R gene duplication event. The functional associations of Clock gene regulation in relation to cis- and trans-effects on gene expression within these genes and additional Clock Controlled Genes within salmonids remains to be investigated.

## Methods

### Mapping panels

Six mapping panels (full-sib families) were used for mapping the *Clock *and *amh *genes. Rainbow trout panels were designated as Lot-25 (n = 48) and Lot-44 (n = 90) [53], Arctic charr panels as AC2 (n = 48) and AC3 (n = 48) [52], and Atlantic salmon panels as BR5 (n = 46) and BR6 (n = 46) [53].

### Sequencing of Clock genes

The *Clock *genes were amplified in five individuals from each of the rainbow trout (Lot25 and Lot44), Atlantic salmon (BR5 and BR6), and Arctic charr (AC2 and AC3) families. To amplify *Clock *in the salmonines, primers were designed from two *Clock *genomic sequences from Chinook salmon *Otsclock1a *(Genbank: DQ780892) and *Otsclock1b *(Genbank: DQ780894). *Clock3 *(Ensembl: ENSDARG00000003631) and *NPAS2 *(Ensembl:ENSDARG00000016536) sequences from zebrafish were used to search the Atlantic salmon BAC End sequences database [121] for putative *Clock3 *and *NPAS2 *sequence. Primers, listed in Tables 1, 2 and 3, were designed to amplify the conserved orthologous regions for *Clock1*, Clock3, and *NPAS2 *copies in rainbow trout, Arctic charr and Atlantic salmon. *amh *sequences were not obtained in this study, but primers designed from known rainbow trout *amh *EST sequences were used to detect SSCP polymorphisms within this gene (see below).

**Table 1:** Primer sequences, purpose, annealing temperature (TA), number of bands and product size for primers during PCR, accession number and the linkage groups (LG) where the fragments were localized in rainbow trout

Primer Name	Primer Sequence (5'→3')		TA	# of Bands Band size	Sequence Name (GB Accession #)	Family & parent	LG	Arm
**Clock1a**	F-GTCCGTCTCTGCTATTGAGATG	Sequencing	61	2	OmyClock1a			
	R-AGTCACCAGCTTAGTCAGCAAC			1.5 kb, 2.5 kb	(GU228520)			
Clock1a-1	F-CCCAGTGTCCAGGTGAGAAA	Mapping	57			44♀♂	8	c
	R-CCTTTGTTCAGGGACACATT							
Clock1a-2	F-ACCCTGGGCATCTTTCTTTT	Mapping	57			25♀♂	8	c
	R-GGAAAACTGTACCATGGGCTA							

**Clock1b**	F-ACCCAGAATACAGGGCAGAAC	Sequencing	51	2	OmyClock1b			
	R-CAGGATCAATTGAGTGGACTGG			1.5 kb, 1.3 kb	(GU228521)			
Clock1b-1	F-AACATGGCCCCTTCCATT	Mapping	60			25♂	24	p
	R-TCTCACCTGGACACTGGACA					44♀	24	p

**Clock3**	F-TTGGAAAGGGTAAATCCTGCT	Sequencing	51	2	OmyClock3			
	R-CTCGCACCTCCGCATAACT			660 bp, 426 bp	(GU228522)			
Clock3-1	F-CACCAGTGGAACTCCAAGC	Mapping	61			25♂	20	p
	R-CGCACCTCCGCATAACTACA							

**NPAS2**	F-TGCAGTTTGGAAAGGGTAAA	Sequencing	51	2	OmyNPAS2			
	R-GAAGTGGCCATGTCAGGTG			602 bp, 487 bp	(GU228523)			
NPAS2-1	F-TTGGAAAGGGTAAATCCTGCT	Mapping	61			25♀	9	p
	R-GCCAGATCTTTCTTCAAGACG							

Amh	F-ACTCCTCCATGTGGTGCAA	Mapping				25♀♂	8	c
	R-CCATACAGTCTTATCTGATCATGC					44♀♂		

**Table 2:** Primer sequences, purpose, annealing temperature (TA), number of bands and product size for primers during PCR, accession number and the linkage groups (LG) where the fragments were localized in Atlantic salmon

Primer Name	Primer Sequence (5'→3')		**T**_**A**_	# of Bands Band size	Sequence Name (GB Accession #)	Family & parent	LG	Arm
Clock1a-1	F-CCCAGTGTCCAGGTGAGAAA	Mapping	57			BR5♀	2	qa
	R-CCTTTGTTCAGGGACACATT							
Clock1a-2	F-ACCCTGGGCATCTTTCTTTT	Mapping	57			BR5♂	2	qa
	R-GGAAAACTGTACCATGGGCTA							

**Clock1b**	F-ACCCAGAATACAGGGCAGAAC	Sequencing	51	2	SsaClock1b			
	R-CAGGATCAATTGAGTGGACTGG			1.5 kb, 1.3 kb	(GU228525)			
Clock1b-2	F-GGTGCAGATGTTCCTCCAA	Mapping	57			BR6♀	18	q
	R-TCACAGAAGACTGTGAGAGGAC							
Clock1b-3	F-GAGTACTGCCCTGCAGGTTG	Mapping	55			BR5♀	18	q
	R-TTGACCATGGCCCTCTTATG							

NPAS2-2	F-CCTCCTGAGGGGGAGAAAG	Mapping	55			BR6♀	5	qb
	R-GAAGTGGCCATGTCAGGTG							

**Table 3:** Primer sequences, purpose, annealing temperature (TA), number of bands and product size for primers during PCR, accession number and the linkage groups (LG) where the fragments were localized in Arctic charr

Primer Name	Primer Sequence (5'→3')		**T**_**A**_	# of Bands Band size	Sequence Name (GB Accession #)	Family & parent	LG
Clock1a-1	F-CCCAGTGTCCAGGTGAGAAA	Mapping	57			3♀	16
	R-CCTTTGTTCAGGGACACATT						

**Clock1b**	F-ACCCAGAATACAGGGCAGAAC	Sequencing	51	2	SalClock1b		
	R-CAGGATCAATTGAGTGGACTGG			1.5 kb, 1.3 kb	(GU228524)		
Clock1b-3	F-GAGTACTGCCCTGCAGGTTG	Mapping	55			3♂	13
	R-TTGACCATGGCCCTCTTATG						

NPAS2-3	F-CTTATAATCTCCACCCAGCACA	Mapping	61			2♀	43
	R-GTCCGTGACAGAGTGCGATA					3♂	20

amh	F-ACTCCTCCATGTGGTGCAA	Mapping				2♀	13
	R-CCATACAGTCTTATCTGATCATGC					3♀	13

Polymerase chain reactions (PCR) consisted of 150 ng of genomic DNA, 1× PCR buffer (Invitrogen), 1.5 mM MgSO4, 0.2-0.4 μM primer mix, 0.2 mM of each dNTP (Fisher Scientific) and 1 U of High Fidelity Platinum *Taq *polymerase (Invitrogen). The thermal cycler conditions were as follows: denaturation at 94°C for 1 min that was followed by 35 amplification cycles of 94°C for 30 s, 48-62°C for 30 s and 68°C for 1-10 min (1 min per Kbp). The amplification products were observed on 1.0-2.0% TBE agarose gel and purified using Qiaquick Gel Extraction Kit (Qiagen) and inserted into pGEM Easy Vector (Promega). The plasmid DNA from six clones for each PCR product was purified (QIAprep Plasmid Miniprep Kit, Qiagen) and sequenced with T7 and Sp6 primers and the Big Dye Terminator Cycle Sequencing Ready Reaction Kit on the ABI 3730 DNA analyzer (Applied Biosystems, Foster City, California, USA).

### Sequence analysis of Salmonine Clock genes

Basic Local Alignment (BLASTN) [122] searches were used to confirm the identity of the *Clock *sequences. Sequence identity values were determined by pairwise comparisons of the salmonine *Clock *sequences using the Jukes-Cantor correction in MEGA 4.0 [123]. Additional nucleotide and protein alignments were generated by hand using BioEdit Sequence Alignment Editor [124]. Rainbow trout, Atlantic salmon, and Arctic charr *Clock *exons were named based on homology to either Chinook salmon *Clock1a *(*Otsclock1a*) exons or zebrafish *Clock *(*DreClock1*; AF133306) exons. *Clock1b *sequences were aligned with *DreClock1 *exons to determine coding regions. Since *Otsclock1b *(DQ780894) from Chinook salmon has only been partially sequenced the exons were not annotated, and thus reference is made to the orthologous exon blocks in zebrafish. NPAS2 exons were determined by alignment with NPAS2 (Ensembl: ENSGACG00000020338) from stickleback. Sequencing and mapping primers for the genes investigated in this study, along with their GenBank accession numbers are provided in Tables 1, 2 and 3 for rainbow trout, Atlantic salmon, and Arctic charr, respectively.

### Phylogenetic analysis of teleost Clock genes

The coding regions from the 3' end of *Clock1 *from medaka (Ensembl: ENSGACG000000; exon 18-21), zebrafish (exon 16-19), Chinook salmon (*Otsclock1a*: exon 16-19; *Otsclock1b*: exon 16-19), rainbow trout (*OmyClock1a*: exon 16-19; *OmyClock1b*: exon 16-19), Atlantic salmon (*SsaClock1b*: exon 16-19), and Arctic charr (*SalClock1a*: exon 16-19), *Clock3 *sequences from zebrafish (Ensembl: ENSDARG00000003631; exon 16-19), and *NPAS2 *sequences from medaka (Ensembl: ENSGACG00000020338; exon 17-20) and zebrafish (Ensembl:ENSDARG00000016536; exon 16-20) were aligned using CLUSTALW [125]. Phylogenetic trees were constructed using the neighbor-joining algorithm for distances and maximum parsimony criteria in MEGA4 [123] at default settings. The MP tree was rooted to the medaka *OlaClock *sequence. The robustness of the tree topology was determined by bootstrap analysis with 1000 replicates.

### Mapping of Clock and amh genes

To localize the *Clock *genes in the rainbow trout, Arctic charr, and Atlantic salmon linkage maps, copy-specific primers were developed to amplify family-based polymorphic insertion-deletion or single nucleotide regions (SNPs) in the sequences (Tables 1, 2 and 3). Anti-müllerian hormone (*amh*) primers were developed from a rainbow trout EST contig cluster TC133387 deposited in the Harvard DFCI gene indices [126]. Polymerase chain reactions were performed in 7 μL reaction volumes consisting of 30 ng of template DNA, 1× PCR buffer (Promega), 0.2 mM each dNTP (Fisher Scientific), 0.1 μM of each primer (one of the primers being 5'-fluorescently end-labeled with tetrachloro-6-carboxyfluorescein (TET)), 2 mM MgCl_2 _(Promega) and 0.15 U of the Go*Taq *DNA polymerase (Promega). The amplifications were performed on a Peltier Thermal Cycler PTC-200 (MJ Research, MA) with the conditions as follows: initial denaturation at 94°C for 4 min followed by 35 amplification cycles of 94°C for 20 s, 48-62°C for 20 s and 72°C for 30 s. The amplification products were run on 6% acrylamide gels for length polymorphisms [31] or single-stranded conformational polymorphism (SSCP) gels for SNPs [8]. The gels were visualized on a FMBIO III scanner and Image Analysis software (Hitachi Genetics Systems). After genotyping the mapping families, LINKMFEX [127] was used to test for deviations from the Mendelian segregation ratio of markers (1:1, log-likelihood G-test) and determine the linkage ordering of markers within specific linkage group assignments using a minimum logarithm of odds score of 4.0.

### Analysis of mapping locations: comparative genomics and QTL

The mapping positions of *Clock *duplicates within a given species were used to indicate homeologous affinities between linkage groups. As well, the mapping positions of *Clock *orthologs across the salmonine species were compared to determine homologies. Comparative synteny analyses between the salmonines and model fish species, zebrafish, stickleback, and medaka, were used to determine the ancient origin of the genomic regions surrounding the *Clock *and *amh *genes. To assess homologies among the three model teleost species, rainbow trout *Clock *and *NPAS2 *sequences were aligned to the genomes of these two species using two approaches. First, BLASTN alignments using the "Distant Homologies" option in http://www.ensembl.org were utilized to assess the most likely contiguous alignments of these sequences to those of the three model species. Second, MEGABLAST [122] was used to search for possible widely gapped alignments to the source model species genomes using the "discontiguous alignments" option. If BLAST alignments suggested homology to a genomic region within any one of the model species that did not correspond to an annotated gene description within the species, the genomic region within that species was further queried for possible gene exon structure using the Softberry program Fgenesh-M [57,128], also contained within the Molquest site[129]. Matches to BLASTN alignments that corresponded to possible exon structure reads with Fgenesh-M were interpreted as possible functional genes that are currently unannotated, while lack of identified exon structure within the region was interpreted as a pseudogene signature. Ancient linkage groups are named as per Kasahara et al. [55] and Danzmann et al. [25]. The mapping locations of the *Clock *genes and *amh *were also compared to previously known life-history and growth quantitative trait loci (QTL) in the salmonine species.

### Synteny analysis of Clock and amh genes

The strand orientation and chromosomal position of genes upstream and downstream to *Clock *and *amh *in zebrafish, medaka, fugu, tetraodon, human, and mouse were determined manually from the gene orientations listed in BIOMART from the Ensembl database [130]. Synteny rearrangements were assessed within homologous linkage group segments using conserved anchor gene positions upstream and downstream of the *Clock *and *amh *mapping locations. Anchor gene positions that were classified as either major sexual development genes or cell-cycling genes were noted.

## Competing interests

The authors declare that they have no competing interests.

## Authors' contributions

Manuscript construction and bioinformatics analyses were preformed by MIP and RGD, while sequencing studies were done by MIP, along with help from HKM. Genotyping analyses were conducted by MIP and HKM. MMF and RGD conceptualized the study. All authors read and commented on the manuscript.

## Supplementary Material

Additional file 1 **Nucleotide sequence alignment of intron 15/16 of *Omyclock1a *and *Salmo salar *Tc1-like transposons DTSsa5**. Click here for file

Additional file 2 **Schematic diagram of conserved syntenic chromosomal regions adjacent to *Clock *and anti-müllerian hormone (*amh*) genes in medaka (*Oryzias latipes*), green-spotted pufferfish (*Tetraodon nigroviridis*), zebrafish (*Danio rerio*), fugu (*Takifugu rubripes*), mouse (*Mus musculus*), and humans (*Homo sapiens*)**. Click here for file
