# Supplementary material for: Clock genes and their genomic distributions in three species of salmonid fishes: Associations with genes regulating sexual maturation and cell cycling
Source: BMC Res Notes. 2010 Jul 29;3:215. doi: 10.1186/1756-0500-3-215 (PMC3161366; doi:10.1186/1756-0500-3-215)
Supplement: Additional file 1 — Nucleotide sequence alignment of intron 15/16 of Omyclock1a and Salmo salar Tc1-like transposons DTSsa5. [file 1756-0500-3-215-S1.PDF]

```
OmyClock1a  1    AAACTCAACA ACAGCAAAAA TTGTCCTCTC ACTATCAACT GCGTTTATTT TCAGCAAACT TAACATGTGT AAATGTTTGT ATGAACATAA
Ssa Tc1     1    AAACTCAGCA A-AAAAAGAA AAGTCCTCAT ACTGTCAACT GCGTTTATTT TCAGCAAACT TAACATGTGC AAATATTTGT ATGAACATAA

OmyClock1a  91   CAAGATTCAA CAACTGAGAC ATACACTG-- ------TCAC TAACA---GA AATTGAATAA TGTGTCCCTG AACAAAGGGG GGGTCAAAA-
Ssa Tc1     91   CAAGATTCAG CAACTGAGAC ATAATCTGAA CAAGTTCCAC AGACATGTGA AATGGAATAA TGCGTCCCTG AACACAGGGG GGATCAAAAT

OmyClock1a  181  -----GTAAC AGTCAGTATC TGGTGTGGCC ACCAGCTGCA TTAAGTACTG CAGTGCATCT CCTCCTCATG GACAGCACCA GATTTGCCAG
Ssa Tc1     181  CAAAAGTAAC AGTCAGTATC TGGTGTGGCC ACCAGCTGCA ---------- ---------T CCTCCTCATG GACTGCACCA GATTTGCCAG

OmyClock1a  271  GTCTTGCTGT GAGATGTTAC CCCACTCTTC CACCAAGACA ACTGCAAGTT CCCAGACATT TCTGGGGGGA ATGGCCCTAG CCCTCGCCCT
Ssa Tc1     271  TTCTTGCTGT GAGATGTTAC CCCACTCTTC CACCAAGGCA CCTGCAAGTT CCCGGGCATT TCGGGGGGGA ATGGCCCTAG CCCTCACCCT

OmyClock1a  361  CCGACCCAAC AGGTCCCAGA CGTGCTCAAT GGGATTGAGA TCCGGGCTCT TTGCTGGCCA TGGCAGAACA CTGACATTCC TGTCTTGCAG
Ssa Tc1     361  CCGATCCAAC AGGTCCCAGA CGTGCTCAAT GGGATTGAGA TCCGGGCTCT TCGCTGGCCA TTGCAGAACA CTAACATTCC TGTCTTGCAG

OmyClock1a  451  GAAATCATGC ACAGAATGAG CCGTGTGGTT GGTGGCATTG TCATGCTGGA GGGTCATATC AGTATGAGCC TGCAGGAAGG GTACCACATG
Ssa Tc1     451  GAAATCATGT ATAGAATGAG CAGTATGGCT GGTTGCATTG TCATGCTAGA GGGTCATGTC AGGATGAGCC TGCAGGAAGG GTACCACATG

OmyClock1a  541  AGGGAGGAGG ATGTCTTCCC TGTAACGCAC AGCGTTGAGA ATGTCTGCTC TGCAATGACA ACAAGCTCAG TCCGAGGATG CTGTGACACA
Ssa Tc1     541  AGGGAGGAGG ATGTCTTCCC TGTAACGCAC AGTGTTGAGA TTGCCT---- -GCAGTGACA ACAAGCTCAG TCCGATGATG CTGTGACACA

OmyClock1a  631  CCGCCCCAGA CCACGACGGA CCCTCCACCT CCAAATCGAT CCCGCTCCAG AGTACAGGCC TCGGTGTAAC GCGCATTCCT TCGAAGATAA
Ssa Tc1     631  CCGCCTCACA CCATGATGGA CCCTCCACCT CCAAATCGAT CTCGCTCCAG AGTACAGGTC TCGGTGTAAC GCTCATTCCT TTGACGATAA

OmyClock1a  721  ACACGAATCC GACCATCACC C-TGGTGAGA CAAAACCGCG ACTCGTCAGT GAAGAGCACT TTTTGCCAGT CCTATCTGGG CCAGTGACGG
Ssa Tc1     721  ACGTGAATCC AACCATCACC CCTGGTGAGA CAAAACCGCG ACTCGTCAGT GAAGAGCACT TTTTGC-AGT CTTGTCTGGT CCAGCGACTG

OmyClock1a  811  TGGGTTTGTG CCCACAGGCG ACATTGTTGC CGGTGATTTC TGGTGAGGAC CTGCCTTACA ACAGGCCTAC AAGCCCTCAG TCCAGCCTCT
Ssa Tc1     811  TGGGGTTGTG CCCATAGGTG ACGTTGCTGC CGGTGATGTC TGGTGAGGAC CTGCCTTACA ACAGGCCTAC AAGCCCTCAG TCCAGCCTCT

OmyClock1a  901  CTCCGCCTAT TGCGAACAGT CTGAGCACTG ATGGAGGGAT TGTGCGTTCC TGGTGTAACT CGGGCAGTTG TTGTTGCCAT CCTGTACCTG
Ssa Tc1     901  CTCAGCCTAT TGCGGACAGT CTGAGCACTG GTGAAGGGAT TGTGCGTTCC TGGTGTAACT CGGGCAGTTG TTGTTGCCGT CCTGTACCTG

OmyClock1a  991  TCCCGCAGGT GTGATGTTCG AATGTATCGA TCCTGTGCAG GAGTTGTTAC ACGTGGTTCT GCCACTGCGT TGATGATCAG CTGTCAGTCC
Ssa Tc1     991  TCCCGCAGGT GTGATGTTCG GATGTACCGA TCCTGTGCAG GTGTTGTTAC ACGTGGT-CT GCCACTGCGA GGACAATTAG CTGTCTGTCC

OmyClock1a  1081 TGTCTCCCTG TAGCGCTGTC TTAGGCGTCT CACAGTACGG ACATTGCAAT TTATTGCTCT GGCTACATCT GCAGTCCTCA TGCCTCCTTG
Ssa Tc1     1081 TGTCTCCCTG TAGCGCAGTC TTAGGCGTCT GACATTACGG ACATTGCAAT TTATTGCCCT GGCCACATCT GCAGTCCTCA TGCCTCCTTG

OmyClock1a  1171 CAGCATGCCT AAGGCAT--- ------GATG AGCAGGGACC CTGGGCATCT TTCTTTTGGT GTTTTTCAGA GTCAGTAGAA AGGCCTCTTT
Ssa Tc1     1171 CAGAATGCAT AAAGCACTTT TACTCAGATG AGCAGGGACC CTGTGCATCT TTTTTTGGGG TGTTTTCAGA GTCAGTAGAA AGGTTTCTTT

OmyClock1a  1261 AGTGTCCTAG GTTTTCATAA CTGTGACCTT AATTGCCTAC CGTCTGTAAG CTGTTAGTGT CTTAACGACC GTTCCACAGG TGCATGTTCG
Ssa Tc1     1261 AGTGTCCTAA ATTTT-ATAA CTGTGACCTT ATTTGCATAC CGTATGTAAC CTGTTAGTAT CTTAACGACC GTTCCACAGG TGCATGTTCA

OmyClock1a  1351 TTAATTGTTT ATGGTTCATT GAACAAGCAT GGGAAACAGT GTTTAAACCC TTTACAATGA AGATCTATGA AGTTATTTGG ATTTTTACGA
Ssa Tc1     1351 TTAATTGTTT ATGGTTCATT GAACAAGCAT GGGGAACAGT GTTTAAACCT TTTACAATGA AGATCTGTGA AGTTATTTGG ACTTTTATGA

OmyClock1a  1441 ATTATCTTTG AAAGACAG
Ssa Tc1     1441 AATTTCTTTG AAAGACAG
```
