# Supplementary material for: Clock genes and their genomic distributions in three species of salmonid fishes: Associations with genes regulating sexual maturation and cell cycling
Source: BMC Res Notes. 2010 Jul 29;3:215. doi: 10.1186/1756-0500-3-215 (PMC3161366; doi:10.1186/1756-0500-3-215)
Supplement: Additional file 2 — Schematic diagram of conserved syntenic chromosomal regions adjacent to Clock and anti-müllerian hormone (amh) genes in medaka (Oryzias latipes), green-spotted pufferfish (Tetraodon nigroviridis), zebrafish (Danio rerio), fugu (Takifugu rubripes), mouse (Mus musculus), and humans (Homo sapiens). [file 1756-0500-3-215-S2.PPT]

## Slide 1
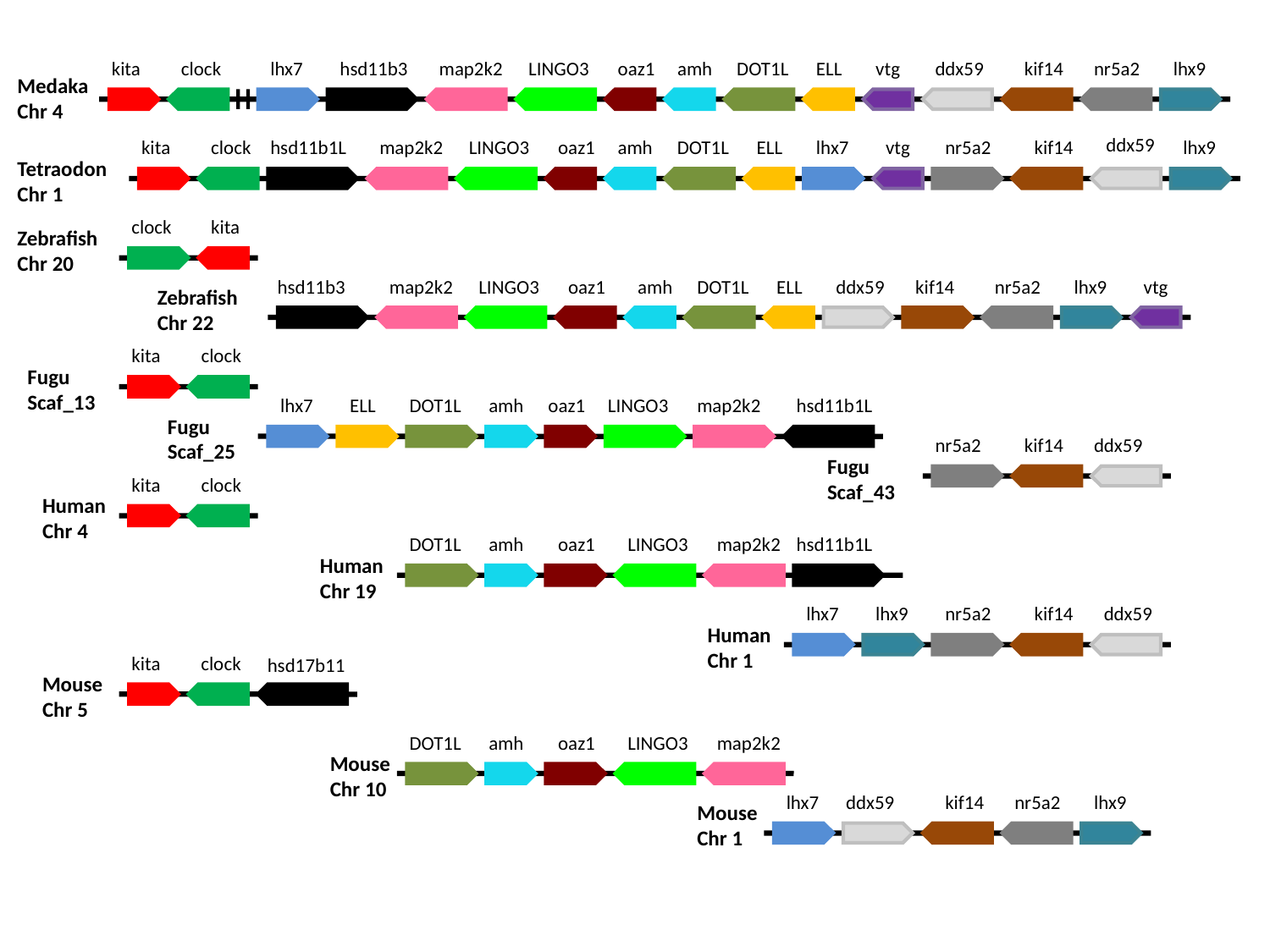

kita
clock
lhx7
hsd11b3
map2k2
LINGO3
oaz1
amh
DOT1L
ELL
vtg
ddx59
kif14
nr5a2
lhx9
Medaka Chr 4
ddx59
kita
clock
hsd11b1L
map2k2
LINGO3
oaz1
amh
DOT1L
ELL
lhx7
vtg
nr5a2
kif14
lhx9
Tetraodon Chr 1
clock
kita
Zebrafish Chr 20
hsd11b3
map2k2
LINGO3
oaz1
amh
DOT1L
ELL
ddx59
kif14
nr5a2
lhx9
vtg
Zebrafish Chr 22
kita
clock
Fugu
Scaf_13
lhx7
ELL
DOT1L
amh
oaz1
LINGO3
map2k2
hsd11b1L
Fugu
Scaf_25
nr5a2
kif14
ddx59
Fugu
Scaf_43
kita
clock
Human
Chr 4
DOT1L
amh
oaz1
LINGO3
map2k2
hsd11b1L
Human
Chr 19
lhx7
lhx9
nr5a2
kif14
ddx59
Human
Chr 1
kita
clock
hsd17b11
Mouse
Chr 5
DOT1L
amh
oaz1
LINGO3
map2k2
Mouse
Chr 10
lhx7
ddx59
kif14
nr5a2
lhx9
Mouse
Chr 1
